# Polyphenols Bioactive Metabolites, and Their Anti-Biofilm and Neuroprotective Potential

**DOI:** 10.3390/foods14223976

**Published:** 2025-11-20

**Authors:** Filomena Nazzaro, Francesca Coppola, Florinda Fratianni, Manar Abdalrazeq, Maria Neve Ombra, Beatrice De Giulio, Raffaele Coppola, Gokhan Zengin

**Affiliations:** 1 Institute of Food Science, CNR, Via Roma 64, 83100 Avellino, Italy; francesca.coppola2@unina.it (F.C.); florinda.fratianni@isa.cnr.it (F.F.); marianeve.ombra@isa.cnr.it (M.N.O.); beatrice.degiulio@isa.cnr.it (B.D.G.); coppola@unimol.it (R.C.); 2 Department of Food Science, University Federico II, Piazza Carlo di Borbone, 1, 80055 Portici, Italy; 3 Q Center, Biomedical Department, Global University College of Science and Health (GUCSH), Rawabi P666, Palestine; m.abdelrazeq@wuhs.edu.bz; 4 Department of Agricultural, Environmental and Food Sciences, University of Molise, Via De Sanctis, 86100 Campobasso, Italy; 5 Department of Biology, Faculty of Science, Selcuk University, 42250 Konya, Turkey; gokhanzengin@selcuk.edu.tr

**Keywords:** polyphenols, gut microbiome, biofilm, quorum sensing, neurodegenerative diseases, neuroprotection

## Abstract

Polyphenols are widely studied phytochemicals with well-known antioxidant and anti-inflammatory properties. They are commonly present in fruits, vegetables, and plant-based foods. Beyond these classical roles, growing evidence shows that polyphenol-derived bioactive metabolites—produced or modified by the gut microbiota—can promote host health. These metabolites are increasingly recognized for shaping host–microbe interactions and influencing neurophysiological functions via the gut–brain axis. This review provides an overview of polyphenol transformation rates by the gut microbiome, highlighting their microbial transformation, anti-biofilm effects, and neuroprotective potential. In our opinion, a deeper understanding of the properties of these metabolites can significantly impact food science and biotechnology.

## 1. Introduction

The human gut microbiota plays a crucial role in host physiology by transforming dietary components into bioactive molecules. Among these, polyphenols are a diverse class of secondary plant metabolites with well-documented antioxidant and anti-inflammatory properties [1]. Increasingly, they are being re-evaluated in the context of host–microbe interactions, which broadens the understanding of their biological significance.

Because polyphenols are poorly absorbed in the upper gastrointestinal tract, they reach the colon largely intact, where they undergo extensive microbial transformation [2]. This process generates low-molecular-weight phenolic metabolites, such as phenolic acids, urolithins, and valeric acids, which often exhibit greater bioactivity and bioavailability than their parent compounds [3]. These metabolites serve as key mediators in the diet–microbiota–host axis, influencing immune, metabolic, and neurological pathways [4,5].

Recent studies highlight their potential as anti-biofilm agents that can attenuate pathogen virulence without promoting antimicrobial resistance [6,7]. Moreover, their ability to modulate the gut–brain axis suggests a role in neuroprotection and the prevention of neurodegenerative diseases such as Alzheimer’s and Parkinson’s [8,9]. This review explores the microbial metabolism of polyphenols, the resulting bioactive metabolites, and their mechanistic roles, with a focus on anti-biofilm and neuroprotective potential. Such information could help design microbiota-friendly foods, including prebiotics, synbiotics, and polyphenol-rich formulations relevant to human health and applied food microbiology.

## 2. Polyphenols: Chemical Classification and Dietary Sources

### 2.1. Chemical Classification of Polyphenols

**Figure 1:**
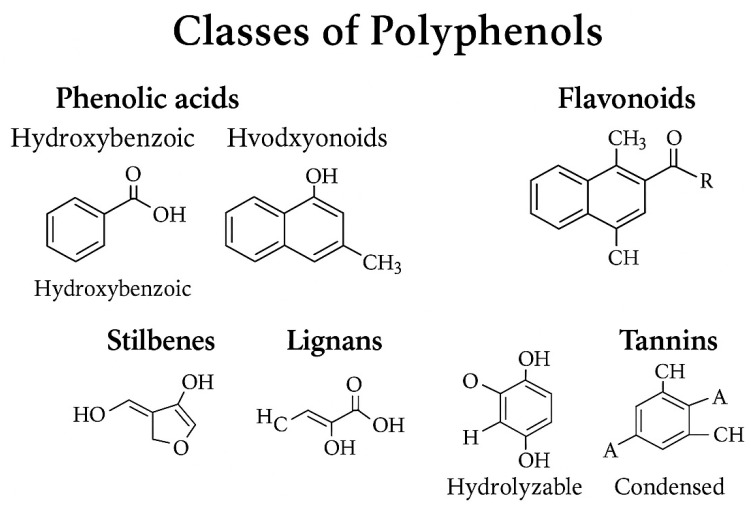
Molecular structure of the main classes of polyphenols.

Polyphenols are chemically heterogeneous plant metabolites defined by one or more aromatic rings with hydroxyl groups (Figure 1). This structural framework underlies their antioxidant activity and their interactions with enzymes, receptors, and microbial communities [6]. Dietary polyphenols are generally classified into phenolic acids, flavonoids, stilbenes, lignans, and tannins [10]. This classification is key to understanding their physicochemical properties, metabolic fate, and health effects (Table 1).

#### 2.1.1. Phenolic Acids

Phenolic acids comprise two primary subclasses: hydroxybenzoic acids, which contain seven carbon atoms, and hydroxycinnamic acids, which contain nine carbon atoms [16]. Specifically, the derivatives of benzoic acid—including gallic, p-hydroxybenzoic, protocatechuic, salicylic, and ellagic acids—serve as antioxidant and antimicrobial agents in plants. In contrast, the derivatives of cinnamic acid—including *p*-coumaric, caffeic, and ferulic acids—act as intermediates in plant metabolic pathways. These compounds are commonly found in berries, coffee, tea, and whole grains, where they often exist as esters or glycosides. Notably, they are typically small, water-soluble molecules that undergo relatively rapid microbial metabolism in the colon, resulting in bioactive derivatives such as di-hydroxy-phenyl-propionic and phenylacetic acids.

#### 2.1.2. Flavonoids

Flavonoids are the largest and most diverse group of polyphenolic compounds found in our diet, with over 6000 types identified in nature. All flavonoids share a basic structure consisting of two six-carbon rings (the A and B rings) joined by a three-carbon link, which usually forms a third, central ring (the C ring). This similar structure allows flavonoids to be grouped into subclasses based on small chemical changes in the central ring. Each type has different features based on how the rings gain or lose small chemical groups, such as sugars, hydroxyl, or methyl groups. Examples of common flavonoids include quercetin, catechin, naringenin, cyanidin–glycoside, and daidzein. Flavonoids can be found as pure compounds or attached to sugars and other molecules. These compounds give fruits, vegetables, herbs, and spices their colours and are also known as dietary supplements that help support health and prevent diseases (10,17). Table 2 shows the main flavonoid subclasses.

#### 2.1.3. Stilbenes

Stilbenes are non-flavonoid polyphenols made of two phenyl rings (A and B) linked by a two-carbon ethylene bridge. They occur as cis or trans isomers and may be free or glycosylated compounds [17]. The A ring usually has two meta-oriented hydroxyl groups. The B ring shows variable hydroxyl or methoxy substitutions. In plants, stilbenes are synthesized as a defence mechanism against pathogens, mainly through the phenylpropanoid pathway. Resveratrol (3,5,4′-trihydroxystilbene) is the most studied of these compounds. It is found in grapes, berries, and peanuts [16]. Resveratrol is abundant in red wine; both it and its microbial metabolites (such as dihydroresveratrol) have strong antioxidant and neuroprotective properties. These properties may lower the risk of cardiovascular and neoplastic diseases [10,18].

#### 2.1.4. Lignans

Lignans are non-flavonoid polyphenols formed by the dimerization of two C6–C3 phenylpropanoid units via a β–β-β′(8–8′) bond. Their structural diversity, due to varied C9 substitutions, gives rise to several subtypes, including furan, dibenzylbutane, and dibenzylbutyrolactone derivatives [19]. Found mainly in legumes, oilseeds (e.g., flaxseed and sesame), and whole grains, lignans occur primarily in the aglycone form. Dietary lignans show antioxidant, estrogenic, and chemopreventive properties [10]. Notably, secoisolariciresinol diglucoside (SDG) is metabolized by gut microbes into enterolignans—specifically, enterodiol and enterolactone—which exhibit phytoestrogenic, anti-inflammatory, and cardioprotective effects, particularly in hormone-dependent cancers [10,19].

#### 2.1.5. Tannins

Tannins are large plant compounds (weighing around 500 to 30,000 Daltons) and come in two main types: condensed and hydrolysable. Condensed tannins are composed of repeated units, such as catechin and epicatechin. A-type tannins have a mix of bonds, while B-type tannins only have strong bonds, making them hard to break down except by gut microbes. Hydrolysable tannins are made when gallic or ellagic acid joins to sugar, forming gallotannins and ellagitannins. Tannins are commonly found in fruits, grains, beans, tea, and wine, where they help protect plants and offer humans antioxidant and anti-inflammatory benefits.

### 2.2. Factors Affecting Polyphenol Content

Genetic, environmental, and technological factors shape the polyphenol content of foods. These factors influence not only concentration but also composition, stability, and bioavailability (Table 3). Post-harvest and processing steps—such as drying, roasting, fermentation, or mechanical disruption—can trigger chemical changes. Sometimes these transformations enhance bioactivity [20,21]. Ripeness and harvest timing alter the balance among anthocyanins, flavonols, and tannins. Agronomic and environmental conditions, such as UV exposure, soil composition, and altitude, regulate phenolic biosynthesis through the phenylpropanoid pathway [22,23,24,25]. These variables ultimately affect the availability of polyphenols as substrates for microbial metabolism, shaping nutritional and functional outcomes.

These factors—processing, maturity, and agronomic environment—determine the quantity and type of polyphenols in the diet, and influence their bioavailability. Furthermore, these same factors directly affect the production of colonic metabolites through microbial fermentation. Given these interconnected effects, the nutritional and functional potential of polyphenols must be assessed holistically. This holistic assessment requires considering their presence in raw materials as well as their fate during food transformation, digestion, and microbial metabolism.

### 2.3. Polyphenol Structural Diversity and Biological Relevance

Polyphenols vary greatly in structure. Hydroxylation, glycosylation, and polymerization determine solubility, stability, metabolism, and bioactivity. Native forms often need microbial or enzymatic conversion into smaller, more absorbable metabolites to exert biological effects [26].

Stilbenes. Resveratrol (trans-3,5,4′-trihydroxystilbene) has potent antioxidant activity but low bioavailability. Microbial conversion to dihydroresveratrol and lunularin may enhance activity. Methylated forms like pterostilbene improve lipophilicity and absorption [27,28,29].

Lignans. Plant lignans such as secoisolariciresinol diglucoside from flaxseed depend on microbial deglycosylation and O-demethylation to produce enterodiol and enterolactone—lipophilic metabolites with estrogenic, antioxidant, and anti-inflammatory properties [30].

Tannins include hydrolyzable (ellagitannins) and condensed (proanthocyanidins) forms. These are large, highly hydroxylated molecules that reach the colon largely intact. There, microbial depolymerization yields catechins, phenolic acids, valerolactones, and urolithins—metabolites with potent systemic effects [26,31,32,33,34,35,36,37,38].

Overall, microbial metabolism converts structurally complex dietary polyphenols into low-molecular-weight polyphenol metabolites (LMWPMs) that mediate their health-promoting properties.

### 2.4. Microbial Transformation of Polyphenols

Polyphenols exhibit a diverse range of biological activities in vitro. However, their in vivo effects largely depend on microbial catabolism. Due to their large size, low solubility, and rapid conjugation (via glucuronidation, sulfation, and methylation), less than 10% of dietary polyphenols are absorbed in the small intestine. Most reach the colon [39]. In the colon, gut microbes convert polyphenols into LMWPMs with a molecular weight of less than 300 Da. These include phenylacetic acids, hydroxyphenylpropionic acids, γ-valerolactones, and urolithins [40,41]. These metabolites exhibit enhanced bioavailability, antioxidant, anti-inflammatory, neuroprotective, and anti-biofilm activities. Key microbial enzymes, such as β-glucosidases, esterases, reductases, decarboxylases, and dehydroxylases, facilitate hydrolysis, reduction, and ring cleavage. These processes produce metabolites that cross biological barriers such as the intestinal mucosa and the blood–brain barrier [42,43,44,45]. Table 4 presents the microbial pathways and the primary enzymes involved in polyphenol metabolism.

Specific taxa, including *Gordonibacter*, *Eggerthella*, *Clostridiales*, and *Slackia*, play a central role in these pathways, generating urolithins, valerolactones, and dihydroresveratrol [37,58,59,60,61]. Distinct classes of polyphenols, such as flavonoids, stilbenes, lignans, and phenolic acids, undergo degradation pathways regulated by specialized microbial enzymes and taxa. For example, ellagitannins are transformed into urolithins by *Gordonibacter urolithinfaciens* and *Ellagibacter isourolithinifaciens*, flavan-3-ols into valerolactones by *Clostridiales* and *Eggerthellaceae*, and resveratrol into dihydroresveratrol by *Slackia equolifaciens* [60]. Variations in gut microbial composition and enzyme activity result in distinct metabotypes, which influence an individual’s ability to metabolize and respond to polyphenols. For instance, some individuals convert isoflavones to equol, while others do not. Comparable differences are observed for ellagitannins, resulting in urolithin metabotypes A, B, or 0, and for resveratrol, as either producers or non-producers of lunularin. Individuals who produce urolithin often demonstrate enhanced cellular energy production, whereas those who produce valerolactone may experience improved cognitive function and reduced inflammation. Thus, microbial breakdown is essential to the physiological effects of dietary polyphenols.

The relationship between dietary polyphenols and the gut microbiota is increasingly viewed as a symbiotic dialogue, rather than a unidirectional interaction. In this dynamic crosstalk, polyphenols are both sculptors and clay—they shape the microbial community while simultaneously being transformed by it. The result is the production of minor, bioactive compounds—postbiotic metabolites—that can exert systemic effects on the host.

This bidirectional relationship can be deconstructed into several interconnected mechanisms: microbial biotransformation, compositional modulation, functional gene enrichment, and feedback amplification. Table 5 shows the function exhibited by specific metabolites produced by the principal microbial genera. All such processes can support the development of personalized nutrition strategies. Further research into these molecules may also inform dietary interventions for individuals with inflammation, infections, or neurological disorders.

### 2.5. Biofunctional Properties of LMWPMs

A growing body of research highlights the biological relevance and pleiotropic activity of LMWPMs. These compounds, which are products of gut microbial metabolism of dietary polyphenols, are emerging as crucial players in managing human health challenges, particularly cardiovascular disease and neurodegenerative disorders. Despite their structural simplicity, LMWPMs exhibit remarkable bio-efficacy across various physiological systems. They often surpass their parent polyphenols in potency, specificity, and systemic availability.

− **Quorum Sensing Inhibition and Anti-Biofilm Activity**

Multiple LMWPMs interfere with quorum sensing (QS), the bacterial communication system that regulates virulence and biofilm development. This includes the disruption of foodborne pathogens. In the complex gut environment, these interactions become even more significant. By disrupting QS mechanisms, LMWPMs can influence not just isolated pathogens, but also the larger microbial consortia in the gut. This may alter microbial dynamics to favor gut health. For example, urolithin A inhibits the *agr* and *sar*A systems in *Staphylococcus aureus.* This attenuates biofilm maturation without affecting planktonic growth. Similarly, 3,4-dihydroxyphenylacetic acid, a flavonoid catabolite, disrupts AI-2–mediated signaling in *E. coli.* This impairs adhesion and EPS synthesis. These activities often occur at sub-inhibitory concentrations. As a result, selective pressure and the risk of antimicrobial resistance are reduced [93,94].

− **Neuroactive Potential and Blood–Brain Barrier Interaction**

Certain LMWPMs can cross the blood-brain barrier (BBB) and interact with neuronal and glial targets, highlighting their potential role in the gut-brain axis. Recognizing the bidirectional signaling between gut microbes and the brain helps us understand how these compounds fit into neural pathways influenced by the microbiota [55,56,57,58,59,60,61,62,63,64,65,66,67,68,69,70,71,72,73,74,75,76,77,78,79,80,81,82,83,84,85,86,87,88,89,90,91,92,93,94,95,96,97]. Furthermore, they can act either directly or by modulating systemic inflammation. For example, dihydroresveratrol exhibits neuroprotective effects by decreasing microglial activation and reducing the expression of TNF-α and IL-6 in the hippocampus. Additionally, urolithin A promotes mitochondrial biogenesis and autophagy (mitophagy) in neuronal models, resulting in enhanced synaptic function and improved memory performance in aging mice. Similarly, some valerolactones modulate monoaminergic signaling, including dopamine and serotonin, suggesting a role in mood and cognitive regulation. Finally, due to their small size, increased lipophilicity, and efficient transporter utilization, LMWPMs cross the blood-brain barrier more readily than their parent polyphenols [97,98].

− **Enhancement of Gut Barrier Function**

Several LMWPMs support intestinal epithelial integrity, which is essential for immune homeostasis and preventing systemic inflammation. Urolithins and phenolic acids upregulate tight junction proteins, including occludin, claudin-1, and ZO-1, in Caco-2 and organoid models. These effects correlate with reduced paracellular permeability and lower endotoxemia in LPS-induced inflammation models. These properties position LMWPMs as potential therapeutic agents for conditions such as increased intestinal permeability, inflammatory bowel diseases (including Crohn’s disease and ulcerative colitis), metabolic syndrome, and neurological disorders [98,99].

− **Epigenetic Modulation and Gene Regulation**

An emerging dimension of LMWPM activity involves epigenetic mechanisms, including inhibition of histone deacetylases (HDACs), which promotes chromatin relaxation and gene transcription, contributing to antioxidant and anti-inflammatory responses. MicroRNA (miRNA) modulation similarly affects apoptosis, cytokine production, and neural plasticity. For example, protocatechuic acid downregulates miR-155 and upregulates miR-146a in macrophages, balancing pro- and anti-inflammatory cytokines [100]. Urolithin B inhibits HDAC1 and HDAC3 in cancer and neurodegeneration models, reactivating neuroprotective genes. Thus, dietary polyphenols and their microbial conversion into LMWPMs are key drivers of these epigenetic shifts, underscoring the synergy between microbiome modulation and gene expression. These activities open new avenues for nutritional epigenetics and postbiotic-based gene modulation strategies [97,98].

### 2.6. Relationship Between Dietary Polyphenols and the Gut Microbiota

**Figure 2:**
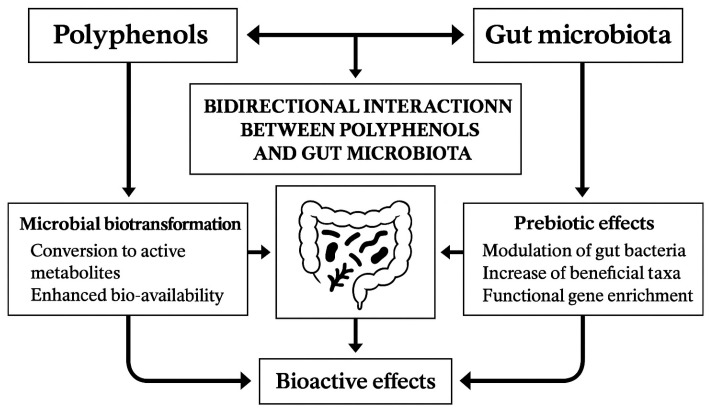
Scenario of the bidirectional interaction between polyphenols and gut microbiota.

The relationship between dietary polyphenols and the gut microbiota is now viewed as a symbiotic dialogue. Polyphenols shape microbial community composition, while the microbiota converts them into smaller, bioactive postbiotic metabolites with systemic effects. This bidirectional exchange involves microbial metabolism of polyphenols, polyphenol-driven modulation of microbiota structure, gene activation, and the cumulative outcomes of these interconnected processes. Table 6 and Figure 2 provide a summary of these processes.

Extensive in vitro and in vivo research shows that polyphenol-rich foods—such as berries, pomegranate, tea, cocoa, and red wine—promote the growth of beneficial gut microorganisms, including *Akkermansia muciniphila*, *Faecalibacterium prausnitzii*, and *Bifidobacterium* spp. Sustained intake of these foods selectively enriches health-promoting taxa. For example, *A. muciniphila*, enhanced by ellagitannin- and flavonol-rich diets, supports gut barrier integrity and reduces metabolic inflammation, while *F. prausnitzii* is stimulated by catechins and chlorogenic acids. *Bifidobacterium* spp., which initiate flavonoid deglycosylation, also benefit from the prebiotic effects of polyphenols and their metabolites, helping maintain eubiosis [46,104]. Anthocyanin intake similarly improves microbiota composition and diversity, promoting the growth of *Bifidobacterium*, *Faecalibacterium*, and *Lactobacillus* [105,106]. Grape polyphenols have been shown to increase *A. muciniphila* abundance and modulate gut barrier parameters in mice [107]. Polyphenol-rich diets also reduce pathobionts such as *Clostridium*, *Fusobacterium nucleatum*, and *Enterobacteriaceae*, contributing to a healthier microbial balance [108,109,110,111].

Polyphenols and their microbial catabolites exert selective antimicrobial activity, suppressing opportunistic and pathogenic taxa at sub-inhibitory concentrations while sparing beneficial commensals [112,113,114,115]. Flavanol- and phenolic acid-rich foods significantly reduce *C. perfringens* and *Enterobacteriaceae*, highlighting their potential to mitigate dysbiosis [45,62]. Microbial metabolites of polyphenols also exhibit selective antimicrobial effects; for instance, the consumption of green tea lowers the levels of faecal *Enterobacteriaceae* and *Clostridium* spp., indicating synergistic prebiotic and antimicrobial actions [116]. Overall, these findings suggest that polyphenols act both as microbial substrates and as modulators of microbial ecology, enhancing their own biotransformation and systemic benefits.

### 2.7. Polyphenol Bioavailability and Microbiota-Driven Biotransformation

Microbial catabolites—produced when gut bacteria break down polyphenols—often exhibit higher absorption, better membrane permeability, and greater biological activity than their parent compounds, substantially contributing to the health benefits of polyphenol-rich diets. The generation of these low-molecular-weight bioactive metabolites depends on both host factors and the gut microbiota.

Among these factors, the composition of the microbiota plays a key role, serving as a primary determinant that links host and dietary influences on bioconversion outcomes. Individual differences in gut microbiota strongly affect polyphenol metabolism. Specific taxa, such as *Gordonibacter*, *Eggerthella*, *Slackia*, and *Adlercreutzia*, catalyze key transformations, including the formation of urolithin and equol, which define distinct metabotypes and bioactive profiles.

Furthermore, in addition to microbial factors, dietary and structural elements also influence the bioconversion process, underscoring the multifactorial nature of polyphenol metabolism. Food matrix (the nutrient and non-nutrient components of food) and polyphenol structure determine how easily polyphenols are accessible to the body and exposed to microbes. Fibers favour colonic (large intestine) metabolism, fats promote early absorption of polyphenols that dissolve in fats (lipophilic compounds), and protein binding lowers availability [108,109,110,111].

In parallel, host physiology further modulates these metabolic processes, integrating internal environmental conditions into the overall metabolic landscape. Colonic pH, transit time, and oxygen influence microbial activity. Longer transit and mildly acidic, anaerobic conditions enhance conversion. High levels of bile acids from diets rich in fat reduce microbial diversity.

Finally, external influences and genetic background are additional determinants that interact dynamically with microbial and host-related factors. Antibiotics suppress key metabolizers (microbes responsible for breaking down polyphenols), while probiotics (beneficial microbes) and prebiotics (food components that support beneficial microbes) enhance the production of short-chain fatty acids (SCFA) and other beneficial metabolites. Age, sex, genetics, and disease states (such as inflammatory bowel disease (IBD) and obesity) influence microbial composition and transformation capacity.

## 3. Anti-Biofilm Activity of Polyphenol-Derived Metabolites

### 3.1. Antimicrobial Resistance and Foodborne Pathogens

Antimicrobial resistance (AMR) is a major global health threat [112,113,114,115,116,117]. Infections caused by antibiotic-resistant pathogens result in greater disease severity and frequent treatment failures in both humans and animals [118,119]. The misuse and overuse of antibiotics in agriculture and the food industry have accelerated the emergence of resistant strains [120], with up to 80% of antibiotics in some countries—such as the United States—used in animal farming [121]. Globally, antibiotic-resistant infections cause an estimated 1.2 million deaths each year [122]. Foodborne pathogens, such as *Campylobacter*, *Salmonella*, *E. coli* O157, *Listeria monocytogenes*, *S. aureus*, and *Clostridium*, infect approximately one in six people worldwide each year [123], often without altering the appearance or sensory qualities of food, which complicates safety assessments [124]. Foodborne pathogens can also cause spoilage and toxin production. These agents contribute significantly to morbidity and mortality. The increased consumption of ready-to-eat foods further exacerbates food safety challenges. Understanding the pathogenic mechanisms of these microorganisms supports advances in epidemiology and detection methods. It also enhances diagnostics and facilitates the development of interventions, including vaccines, pharmaceuticals, and bioactive molecules—such as polyphenols and their metabolites—for the prevention and control of diseases.

### 3.2. Pathogenic Biofilms

Biofilms are structured microbial communities embedded in a self-produced extracellular polymeric substance (EPS) matrix that adheres to various surfaces [125]. These consortia demonstrate significant resistance to antimicrobial agents, host immune defenses, and environmental stressors, which present major challenges in both clinical and food safety contexts. Bacteria within biofilms can survive antimicrobial concentrations up to 1000 times higher than those required to inhibit planktonic cells, thereby contributing to persistent infections [126]. Polyphenols and their derivatives exhibit broad-spectrum antimicrobial activity [127,128,129]. Their microbial metabolites exert multifaceted, non-lethal effects by targeting signaling pathways and structural elements essential for biofilm development, resulting in the destabilization of biofilms, increased dispersal, and heightened susceptibility to antimicrobials. Polyphenols also inhibit quorum sensing, disrupt cellular membranes, suppress enzymatic activity, induce reactive oxygen species (ROS), modulate immune responses, and block viral entry and replication. These mechanisms collectively enable polyphenols to eliminate microbial species. For instance, enzyme inhibition impairs metabolism, membrane disruption leads to cell death, ROS generation causes oxidative damage, quorum-sensing interference affects gene regulation and biofilm formation, and immune modulation strengthens host defense. The precise mechanisms depend on the microorganism, metabolite structure, and environmental conditions. Notably, membrane disruption is a principal antibacterial action of polyphenols [128,130]; interaction with lipid bilayers alters membrane permeability, damages membrane proteins, generates ROS, and ultimately results in cell lysis. Consequently, polyphenols are regarded as effective antifungal, antiviral, and antibacterial agents, and remain central to anti-infective research [127,131,132]. Furthermore, microbial-derived metabolites, such as urolithins, valerolactones, phenylpropionic acids, and dihydrocaffeic acid, frequently demonstrate greater anti-biofilm activity than their parent compounds [129,133].

These metabolites are produced through gut microbial transformation of dietary polyphenols. They frequently demonstrate enhanced bioavailability, stability, and tissue penetration. At sub-inhibitory concentrations, these compounds impair biofilm formation, maturation, or maintenance. They do this without compromising bacterial viability. Consequently, they reduce selective pressure for resistance and help preserve commensal microbial balance.

Microbial-derived polyphenol metabolites have broad-spectrum anti-biofilm activity. They target both Gram-positive and Gram-negative bacteria, as well as fungi. These metabolites interfere with quorum sensing and inhibit cellular adhesion. They also disrupt the extracellular polymeric substance (EPS) matrix and suppress expression of virulence genes. These mechanisms are especially relevant in the context of increasing antimicrobial resistance [126]. Microbial degradation of tea catechins produces several metabolites. These include 3-O-methyl gallic acid, gallic acid, caffeic acid, 4-hydroxyphenylpropionic acid, phenylpropionic acid, and 4-hydroxyphenylacetic acid. All demonstrate antibacterial properties [87]. Urolithin A also shows significant antibacterial activity. It inhibits AHL-mediated quorum sensing, biofilm maturation, and bacterial motility [127,134,135].

### 3.3. Mechanisms of Action of Polyphenol-Derived Metabolites on Foodborne Biofilms

Polyphenols and their derivatives disrupt microbial membranes, compromising integrity or inducing cell death [136,137]. By inhibiting fatty acid biosynthesis through targeting FabG and FabI, EGCG and related compounds exert their effects. In contrast, galloyl groups enhance interactions with lipid bilayers, while catechins penetrate the hydrophobic core of phospholipid membranes, altering their structure [138,139]. Wang et al. [140] demonstrated that antibacterial potency increases with alkyl chain length and that EGCG causes nanoscale membrane damage in *E. coli* and *S. aureus*. Further studies linked *S. aureus* cell wall degradation to EGCG binding to peptidoglycan, while *E. coli* damage is associated with oxidative stress [141]. Together, these findings demonstrate distinct mechanisms against Gram-positive and Gram-negative bacteria [142].

Polyphenols also inhibit the aggregation of amyloid-like proteins, a key structural component of biofilms. Compounds such as quercetin, myricetin, EGCG, and PGG prevent amyloid fibrillation by stabilizing early oligomers, redirecting aggregation pathways, and weakening matrix integrity, thereby increasing susceptibility to antibiotics without affecting bacterial viability [143,144].

Polyphenol-derived metabolites physically interfere with biofilm matrix formation by altering surface properties. By increasing electrostatic repulsion, hydrophilicity, and steric hindrance, they reduce bacterial adhesion and prevent the early establishment of biofilms [145]. Their negatively charged groups enhance surface charge, forming thin molecular layers (~10–20 nm) that act as barriers to attachment. Increased hydrophilicity further decreases bacterial affinity, reducing colonization by *S. aureus* and *E. coli*. These electrostatic and steric effects effectively inhibit biofilm formation in vitro and in vivo, including cell culture and wound models [146,147,148,149,150,151].

### 3.4. Quorum Sensing Modulation

Polyphenol-derived metabolites interfere with bacterial communication, known as quorum sensing, through several mechanisms. These metabolites degrade chemical signals, including acyl-homoserine lactones (AHLs) in Gram-negative bacteria and autoinducing peptides (AIPs) in Gram-positive bacteria, thereby reducing their half-lives from hours to minutes and preventing the activation of genes associated with biofilm formation. Additionally, polyphenol metabolites compete for signal-binding sites, block receptor activation, and inhibit the expression of quorum-sensing–regulated genes, thereby decreasing the production of virulence factors and biofilm formation. Compounds such as tea polyphenols and paeonol can also enhance antibiotic efficacy; for instance, catechins reduce the minimum inhibitory concentration (MIC) of oxacillin against *S. aureus*, demonstrating synergistic therapeutic potential. Disruption of these signalling pathways diminishes bacterial motility, virulence, and biofilm-forming ability, increasing susceptibility to host immune defences and antimicrobial agents. Furthermore, polyphenol metabolites may influence communication between bacteria and host cells, thereby affecting biofilm development and modulating immune responses.

### 3.5. Polyphenol-Derived Metabolites Most Effectively Target Which Food-Borne Pathogens?

Polyphenol-derived metabolites target pathogens, including *E. coli* O157, *S. aureus*, *Salmonella Typhimurium*, *Bacillus cereus*, *L. monocytogenes*, *Enterococcus faecalis*, and *Enterobacter sakazakii*. Polyphenols, such as baicalein, myricetin, hesperetin, and quercetin, can act as bacteriostatic or bactericidal agents, depending on the strain and structure.

***E. coli* O157**: polyphenol-derived metabolites, such as phloretin from apples, downregulate the curli genes (*csgA* and *csgB*), thereby reducing bacterial attachment and weakening the biofilm structure [152].

***S. aureus*** (including MRSA): Tannic acid increases IsaA protein levels, inhibiting biofilm maturation. Hydroxyl groups in flavonoid metabolites disrupt quorum sensing by binding to DNA and proteins, reducing the expression of virulence factors. These metabolites also enhance the effectiveness of antibiotics, such as oxacillin, by weakening biofilm defences, including the *agr* system [94].

***Salmonella:*** polyphenol metabolites inhibit biofilm formation by disrupting the synthesis and motility of extracellular substances [153,154]. Biotin-tagged derivatives target biofilm stability and enhance the efficacy of ciprofloxacin. Some metabolites may downregulate T3SS regulators, limiting biofilm and invasion; further studies are needed. EGCG blocks quorum sensing and the type III secretion system (T3SS), affecting motility and biofilm development. More validation is required for hydroxyphenylvaleric acids and valerolactones [155,156,157,158].

***Enterobacter sakazakii***: various polyphenolic compounds reduce biofilm formation and virulence gene expression. Coenzyme Q10 downregulates biofilm and virulence genes, reducing adhesion and motility. Chlorogenic acid disrupts membrane potential, ATP production, pH balance, and cellular morphology [159,160,161].

***Vibrio parahaemolyticus:*** this microorganism shows resistance, prompting the study of plant-derived compounds. Flavonoids disrupt bacterial membranes, while albofungin damages biofilms by affecting cell structures [162,163,164].

***Enterococcus faecalis***: terpenoid and flavonoid metabolites suppress virulence genes (esp, gelE), reduce biofilm adhesion, and inhibit the release of extracellular DNA needed for stability. Curcuminoids reduce biofilm biomass by more than 70% at sub-MIC doses [165].

***B. cereus***: Polyphenol-derived acids like gallic acid disrupt its membrane potential and inhibit spore and biofilm formation.

***L. monocytogenes***: This pathogen contains key virulence gene clusters, including LIPI-1 and the InlA/InlB operon, essential for invasion and survival [166]. Polyphenol-derived metabolites negatively modulate these genes by inhibiting activation of virulence factors, altering surface proteins, inducing oxidative stress, and affecting quorum sensing, reducing adhesion, invasion, and biofilm formation [166,167,168,169].

### 3.6. Anti-Biofilm Activity of Polyphenol-Derived Microbial Metabolites

Microbial metabolites of dietary polyphenols inhibit biofilm formation in Gram-positive and Gram-negative foodborne bacteria. Their mechanisms include quorum-sensing interference, prevention of adhesion, EPS disruption, and suppression of virulence genes. These features are especially relevant given the rising prevalence of antimicrobial resistance and the need for non-bactericidal strategies [170,171,172]. For example, ellagitannins create hydrolyzed metabolites that inhibit AHL synthases, demonstrating anti-QS effects [173].

Urolithins A and B can impair the motility and adhesion of *L. monocytogenes* on food-processing surfaces, potentially reducing biofilm formation [174]. They also inhibit quorum-sensing systems in pathogens such as *Yersinia enterocolitica*, *Shigella dysenteriae*, *Vibrio cholerae*, *Campylobacter* spp., and MRSA by lowering AHL levels and blocking motility and maturation pathways [50,134,135,143]. Urolithin A additionally reduces *C. difficile* toxin expression without killing cells, indicating a gene-level effect. Several urolithins and derivatives show distinct antibacterial activities. Valerolactone metabolites, such as 5-(3′,4′-dihydroxyphenyl)-γ-valerolactone and its conjugates, show strong anti-adhesive effects against uropathogenic *E. coli* (UPEC), with sulfated derivatives most active at 100 µM [49]. Other microbial metabolites of tea catechins, including 3-O-methyl gallic acid, gallic acid, caffeic acid, phenylpropionic acid, and 4-hydroxyphenylacetic acid, also have antibacterial properties [87]. Compounds like 3,4-dihydroxyphenylacetic acid inhibit UPEC adhesion in a concentration-dependent way [175], and 3,4-dihydroxyphenylpropionic acid may modulate *E. coli* growth dynamics [81].

Phenyl-lactic acid (PLA) derivatives inhibit biofilms by disrupting motility, chemotaxis, quorum sensing, and polysaccharide production, as well as reducing respiratory chain activity. They can also compromise membrane integrity in *S. aureus* and *S. enteritidis*, leading to ATP leakage [176]. Hydroxyphenylacetic acid and 3,4-DHPA sensitize *Salmonella* to novobiocin [177], and related metabolites can reduce *E. coli* resistance to β-lactams and aminoglycosides [178].

### 3.7. Synergy with Antimicrobials

The increasing prevalence of biofilm-associated infections and antimicrobial resistance (AMR) has sparked significant interest in combination therapies that enhance the effectiveness of conventional antibiotics. Polyphenol-rich plant extracts and their microbial metabolites have shown synergistic or additive effects with various antimicrobial agents, especially against biofilm-forming pathogens. These combinations can reduce antibiotic dosages, restore activity against resistant strains, and potentially shorten treatment durations or decrease side effects [179,180].

**Figure 3:**
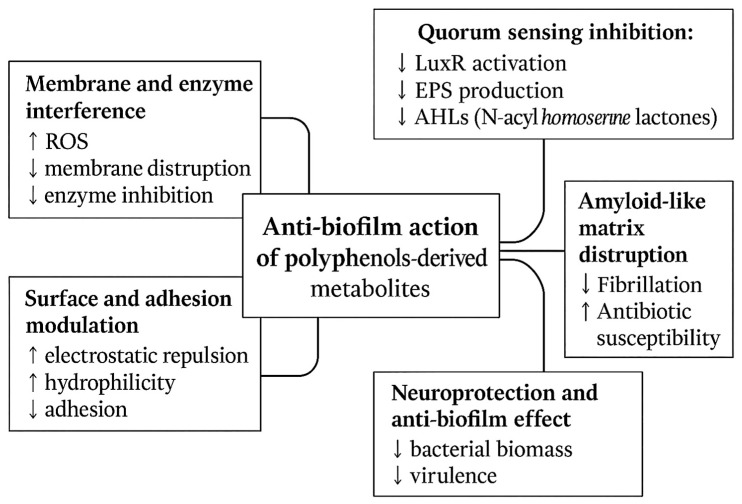
Scheme of convergent mechanisms used by polyphenol-derived metabolites against pathogenic bacteria: Quorum sensing inhibition (blocking bacterial communication); Amyloid-like matrix inhibition (interfering with Bap/FapC and increasing antibiotic penetration); Surface and adhesion modulation (enhancing electrostatic repulsion and hydrophilicity, reducing adhesion); Synergy and sensitization (lowering biomass and virulence, increasing antibiotic susceptibility). Captions: AHLs = N-acyl homoserine lactones; LuxR = quorum-sensing transcriptional regulators; EPS = Extracellular Polymeric Substances; ROS = Reactive Oxygen Species.

**Figure 4:**
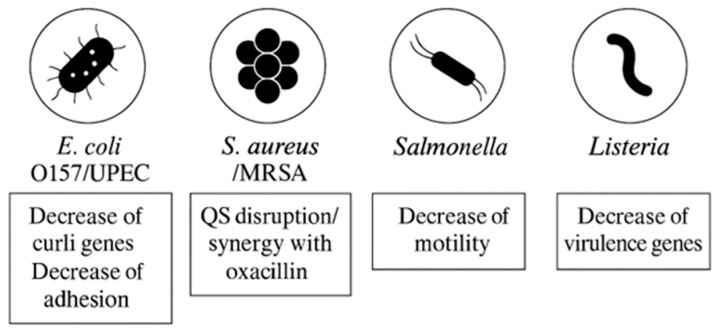
Overview of some representative foodborne pathogens (*E. coli* O157/UPEC, Methicillin-resistant *S. aureus* (MRSA), *Salmonella*, *Listeria*) with dominant antibiofilm actions (anti-adhesive, anti-QS, anti-matrix, membrane disruption, antivirulence) by polyphenol-derived metabolites.

Examples of synergistic combinations: Polyphenols derived from green propolis, *Tabebuia avellanedae* bark, and *Olea europaea* leaf extracts demonstrate synergistic activity with macrolide antibiotics, including azithromycin and clarithromycin, against *S. aureus*. This synergy results in reduced minimum inhibitory concentration (MIC) and minimum bactericidal concentration (MBC) values, as well as enhanced bactericidal effects in time-kill assays [179,180,181]. In extension to these findings in Gram-positive bacteria, combinations such as colistin with the adjuvant PFK-158 have also been shown to synergistically inhibit biofilm formation in Gram-negative bacteria, indicating that comparable polyphenol-based strategies may be effective in this context as well [182]. Therefore, these results collectively support the application of polyphenol-rich extracts and their metabolites, alongside antibiotics, to manage chronic and biofilm-associated infections, resensitize resistant strains, and reduce selective pressure [183]. Furthermore, as polyphenols primarily target bacterial communication and physiology rather than viability, they may also contribute to the preservation of commensal microbiota. Table 7 and Table 8 and Figure 3 and Figure 4 summarize the anti-quorum-sensing and antibiofilm actions of polyphenol-derived metabolites.

## 4. Neuroprotective Properties of Polyphenol-Derived Metabolites and the Gut–Brain Axis

Metabolites produced from dietary polyphenols by gut microbiota act on both pathogenic bacteria and the central nervous system. These bioavailable compounds have antimicrobial and anti-biofilm properties, including effectiveness against foodborne pathogens that cross the intestinal and blood–brain barriers. They also modulate neuroinflammation and offer antioxidant and neuroprotective benefits. Together, these actions make polyphenol-derived metabolites important mediators connecting gut health, infection control, and neurodegenerative processes.

### 4.1. The Gut–Brain Axis and Foodborne Pathogens

The gut–brain axis (GBA) is a bidirectional communication network that links the gut and brain through various pathways, including immune, hormonal, neural, and microbial pathways. This system regulates digestion, immunity, and behavior, but can be disrupted by foodborne pathogens, which trigger gastrointestinal disease and neuroinflammation. Pathogens induce gut immune responses, releasing cytokines that affect mood, cognition, and stress responses [186]. Chronic inflammation from persistent infections can contribute to disorders such as IBS, where altered gut activity influences brain function. By disturbing microbial diversity, pathogens create dysbiosis that transmits abnormal signals to the brain, potentially leading to anxiety, depression, or cognitive changes. Infections such as *Salmonella* or *Clostridium difficile* can also impair neurotransmitter production, including serotonin, leading to nausea, anxiety, and long-term behavioral effects [187,188,189]. Increased gut sensitivity resulting from infection generates pain signals transmitted to the brain, contributing to the development of functional gastrointestinal disorders [190].

The GBA affects immunity and drug metabolism by altering how cells respond to stress caused by pathogen-driven inflammation and microbial imbalances. These changes can reduce the effectiveness of chemotherapy. For example, *Salmonella*’s virulence factors can alter host chromatin, disrupt DNA methylation and histone modification, and activate NF-κB and STAT3, all of which contribute to cancer progression and treatment resistance [191]. Non-coding RNAs, such as miR-21, miR-155, and lncRNAs, regulate cell growth, death, and drug removal, suggesting potential treatments, including probiotics or drugs targeting epigenetic enzymes (HDACs, DNMTs) [192].

Foodborne infections—bacterial, parasitic, or viral—pose a significant global health threat and can elicit neuroinflammatory responses, despite primarily affecting the gut [193]. Key mechanisms include GBA-mediated reshaping of the microbiota, which generates systemic inflammation affecting organs such as neuronal tissues [194,195]. Pathogens can compromise intestinal integrity, known as “leaky gut,” allowing microbes and inflammatory mediators to access the brain [196]. Bacterial metabolites and toxins may accumulate in brain tissue, damage the blood–brain barrier (BBB), and activate cytokine-driven neuroinflammation [197]. These processes link foodborne pathogens to chronic neurological conditions, including brain abscesses, meningoencephalitis, Alzheimer’s disease, Parkinson’s disease, and rhombencephalitis, partly through the effects of neurotoxins and mycotoxins that disrupt BBB integrity [198,199].

### 4.2. Potential Mechanism of Pathogen-Associated Neurotoxicity

Many human diseases compromise brain tissue, and bacterial infections may cause substantial morbidity and mortality of the central nervous system (CNS), resulting in recurrent neurological disorders even in the presence of antibiotic therapy [200]. These outcomes occur in disorders transmitted via various routes, including gastrointestinal infection. For instance, toxins secreted by foodborne pathogens such as *C. botulinum* can induce encephalopathy [201].

### 4.3. Foodborne Pathogens and Their Neurological Impact

*E. coli* and neuroinflammation. Certain strains of *E. coli* (particularly enterohemorrhagic EHEC) produce Shiga toxin, which can penetrate the intestinal epithelium and enter the bloodstream [202]. Once within the circulation, Shiga toxins can traverse the BBB, triggering inflammation and neurodegeneration [186,203]. Pathogen-induced inflammation activates microglia—the immune cells of the nervous system—resulting in chronic oxidative stress and neuronal injury [204].

*Salmonella enterica* and brain inflammation. Although *Salmonella* infections typically affect the intestinal tract, they can disseminate systemically, causing meningitis, particularly in individuals with compromised immune systems. Bacterial lipopolysaccharides (LPS) activate Toll-like receptors (TLRs) in the nervous system [205], promoting the release of pro-inflammatory cytokines such as IL-1β, TNF-α, and IL-6 [206]. This inflammatory cascade can contribute to both cognitive decline and neurodegenerative diseases.

*L. monocytogenes* and meningitis. One of the most important foodborne pathogens affecting the CNS is *L. monocytogenes*, which causes listeriosis. This bacterium can penetrate the intestinal barrier, enter the bloodstream, and cross the BBB to induce encephalitis and meningitis. The potent immune-mediated response elicited by *L. monocytogenes*, involving microglial activation and increased cytokine production, can lead to chronic neuroinflammation and neuronal degeneration [207].

*C. botulinum* and neurotoxicity. Botulinum neurotoxin (BoNT), produced by *C. botulinum*, is among the most potent neurotoxins known. Although BoNT is primarily associated with muscular paralysis, emerging evidence suggests it may also interfere with neuronal communication pathways. In the context of chronic neurodegenerative conditions, even low-dose exposure to *C. botulinum* toxins can provoke mild neuroinflammatory effects [208].

### 4.4. Neurotoxic Effects of Foodborne Toxins

The gut–brain axis plays a crucial role in regulating neuroinflammation [209]. Neurotoxins and metabolites produced by foodborne microbes can compromise the BBB integrity and activate glial cells within the cerebral cortex, triggering neuroinflammation and disrupting neurotransmission [210]. The severity of these neurological effects is influenced by both toxin concentration and the host’s physiological response [186]. For instance, *L. monocytogenes* secretes Listeriolysin O (LLO), which traverses the BBB via circulating cells and interacts with receptors such as InlA, InlB, and Vip, resulting in conditions including meningitis, encephalitis, brain abscesses, and rhombencephalitis [199,211]. Similarly, *C. botulinum* neurotoxin readily crosses the BBB and disrupts the gut–brain axis, leading to neuromuscular paralysis [212]. Additionally, Shiga toxin produced by *E. coli* exacerbates neuroinflammation by increasing cytokine production, activating Toll-like receptors, and causing neuronal damage after crossing the BBB [213,214].

### 4.5. Foodborne Pathogens and BBB Disruption

BBB consists of endothelial cells joined by tight junctions (including claudins, occludin, and ZO-1). It is supported by pericytes, astrocytic end-feet, and the basement membrane, collectively forming the neurovascular unit (NVU). The NVU regulates the exchange of nutrients, metabolites, and immune cells between the circulation and the CNS, while also protecting the brain from toxins and pathogens. Multiple foodborne microbes have evolved mechanisms to breach the BBB, resulting in severe neurological conditions such as meningitis and encephalopathy. The principal pathways include: (i) lipopolysaccharide (LPS)-mediated inflammation, whereby bacteria, such as *E. coli*, *Salmonella*, increase endothelial permeability and disrupt tight junctions [215,216]; (ii) transcellular and paracellular invasion, as demonstrated by *E. coli* K1 receptor-mediated transcytosis [217] and *L. monocytogenes* traversing between endothelial cells [218,219]; (iii) the “Trojan horse” mechanism, in which infected macrophages transport pathogens across the BBB, as observed with *Listeria* and *Salmonella* [186]; and (iv) direct endothelial interaction, exemplified by *Streptococcus suis* binding to host factors to penetrate the BBB [220].

Compromise of the BBB permits the infiltration of toxins, pathogens, and immune cells into the brain, which promotes neuroinflammation, neurodegeneration, and cognitive decline. Disruption of tight junction integrity initiates glial activation and the release of cytokines (TNF-α, IL-6, IL-1β), while oxidative stress and mitochondrial dysfunction further impair barrier function. Chronic foodborne infections can accelerate τ and β-amyloid accumulation in Alzheimer’s disease [221,222], increase BBB permeability and α-synuclein aggregation in Parkinson’s disease [223], and facilitate autoimmune responses such as demyelination in multiple sclerosis, as observed in *Campylobacter jejuni*–induced Guillain–Barré syndrome [224]. Comparable mechanisms may exacerbate vascular injury in stroke, amyotrophic lateral sclerosis (ALS), and other neurological disorders [225]. Collectively, these findings suggest that Gram-negative foodborne pathogens contribute to both gastrointestinal and chronic neurological diseases by disrupting the BBB and altering receptor interactions, underscoring the importance of preventive strategies. Table 9 summarizes the pathogens involved in CNS and neurodegenerative diseases.

### 4.6. Role of Polyphenols and Their Metabolites in Central Nervous System Protection

Disruption of BBB, frequently initiated by pathogenic agents, contributes to neurological disorders including Alzheimer’s disease, Parkinson’s disease, multiple sclerosis, and Huntington’s disease. These conditions are characterized by protein aggregation, mitochondrial dysfunction, oxidative stress, and neuroinflammation [225,226,227]. Native polyphenols exhibit limited central nervous system activity due to poor bioavailability and low BBB permeability. In contrast, microbial-derived metabolites such as urolithins, phenyl-γ-valerolactones, and dihydroxylated phenolic acids demonstrate greater stability, enhanced absorption, and improved BBB penetration [228]. These metabolites reinforce tight junction integrity, attenuate oxidative and inflammatory stress, support mitochondrial quality control, and regulate proteostasis pathways [229,230]. Their colonic production and unique pharmacokinetic profiles contribute to the neuroprotective effects observed with polyphenol-rich diets, highlighting the microbiota as a critical mediator between nutrition and brain health. Metabolites, including urolithin A, phenyl-γ-valerolactones, and dihydroxylated phenylacetic acids, are capable of crossing the BBB, modulating neurocognitive pathways, and exhibiting antibacterial activity against foodborne pathogens. The combined antimicrobial and neuroprotective properties of these compounds may contribute to the prevention or mitigation of central nervous system disorders.

### 4.7. Core Neuroprotective Mechanisms

Anti-inflammatory and antioxidant actions. Both conjugated and free microbial phenolic acids, including 3,4-dihydroxyphenylacetic acid, protocatechuic acid, and dihydrocaffeic acid, as well as their sulfate and glucuronide conjugates, protect human neuronal (SH-SY5Y) cells from LPS- or t-BHP-induced injury. These compounds attenuate reactive oxygen species (ROS) and reduce cytokine production in macrophage and microglial models. Phase-II conjugates often demonstrate greater efficacy at physiologically relevant concentrations [231].

Proteostasis and aggregate toxicity. Phenyl-γ-valerolactones (PVLs)—signature colonic metabolites of flavan-3-ols—modulate cellular proteolytic systems and lower Aβ1-42 levels in neuronal cells; related PVLs selectively detoxify Aβ oligomers and prevent memory impairment in an AD mouse model, pointing to aggregate-focused neuroprotection [230,232].

Mitochondrial quality control (mitophagy) and cellular stress adaptation. Urolithin A acts as a potent inducer of mitophagy and cellular housekeeping pathways across multiple tissues. Preclinical studies in the central nervous system (CNS) demonstrate reduced neuroinflammation, improved blood–brain barrier (BBB) integrity, and protection against neuronal apoptosis following brain injury. These mechanisms are relevant to both acute and chronic neurodegenerative conditions [229,233].

Cerebrovascular function and BBB integrity. PVLs and related metabolites are associated with improved endothelial function and cerebral perfusion in both preclinical and translational studies. Urolithin A (UA) has been shown to directly preserve BBB structure and function in traumatic brain injury models. This mechanism is highly relevant to Alzheimer’s disease and vascular cognitive impairment [229,233].

### 4.8. Alzheimer’s Disease (AD)

BBB disruption and cerebrovascular dysfunction are now considered early hallmarks of AD. These processes contribute to impaired Aβ clearance and neuroinflammation [234]. Microbial polyphenol metabolites influence several AD-relevant pathways. Phenyl-γ-valerolactones (PVLs) reduce amyloid oligomer toxicity by binding to soluble Aβ species. They also support proteostasis by modulating the ubiquitin–proteasome system and autophagy. This lowers intracellular Aβ1-42 accumulation [230,232]. Urolithin A (UA) enhances autophagy and mitophagy, preserves mitochondrial function, and protects BBB integrity. In brain injury models, UA reduces apoptosis and counters AD-related vascular dysfunction [96,229]. Microbial phenolic acids (such as 3,4-dihydroxyphenylacetic and protocatechuic acid) decrease oxidative stress, microglial activation, and inflammatory cascades that drive Aβ aggregation and tau pathology [231]. Epidemiological studies link higher flavonol intake to reduced AD risk. Evidence suggests that gut-derived metabolites—not parent flavonoids—are the primary mediators, highlighting the importance of individual metabotypes [234]. While preclinical data are strong, clinical studies are lacking. No trials have tested purified polyphenol metabolites in AD, though early work on equol and UA shows feasibility. Future AD studies should stratify participants by metabotype, track metabolite exposure, and use neuroimaging and cognitive measures to clarify causality [235].

### 4.9. Parkinson’s Disease (PD)

Microbial-derived polyphenol metabolites affect key PD processes. Urolithin A induces mitophagy, clearing dysfunctional mitochondria and reducing oxidative stress to support the survival of dopaminergic neurons [96]. PVLs enhance cellular proteostasis, reduce protein aggregation, and dampen oxidative and inflammatory responses, mechanisms relevant to both AD and PD [230]. Phenolic acids, including dihydrocaffeic acid and 3,4-dihydroxyphenylacetic acid, protect neurons by inhibiting microglial activation and oxidative injury and influence neurotransmitter metabolism and gut–brain signaling [231]. Epidemiological data link polyphenol-rich diets, such as the Mediterranean diet, to lower PD risk and slower progression. Neuroprotection largely depends on the gut microbiota’s ability to generate UA, PVLs, and other metabolites, highlighting interindividual variability in metabolic responses [236].

### 4.10. Multiple Sclerosis (MS)

Polyphenol-derived microbial metabolites may modulate neuroinflammation in MS. Dihydrocaffeic and 3,4-dihydroxyphenylacetic acids reduce cytokine release, inhibit NF-κB, and limit oxidative stress in glial and immune cells, helping protect myelin and oligodendrocytes [231]. UA supports mitochondrial quality control and BBB integrity, which may enhance resilience to inflammation-induced neuronal damage [229]. Clinical data are lacking, but observational studies suggest that polyphenol-rich diets, such as the Mediterranean diet, may reduce relapse rates and disease progression, possibly through microbiota-dependent mechanisms [237].

### 4.11. Huntington’s Disease (HD)

Polyphenol metabolites may alleviate HD pathology by reducing protein misfolding and mitochondrial dysfunction. PVLs enhance proteolytic systems and limit the accumulation of misfolded proteins [230]. UA promotes mitophagy, improves mitochondrial bioenergetics, and reduces ROS, countering neuronal vulnerability [116]. Microbial phenolic acids, with their antioxidant and anti-inflammatory properties, may also reduce excitotoxic and oxidative injury, which are central to HD progression.

### 4.12. Vascular Cognitive Impairment and Mixed Dementias

VCI, which often overlaps with AD, stems from cerebrovascular dysfunction that worsens cognitive decline [238]. Notably, microbial-derived polyphenol metabolites can support vascular and cognitive health via endothelial and anti-inflammatory effects. Specifically, phenolic acids (e.g., 3,4-dihydroxyphenylacetic, protocatechuic acid) improve endothelial function, reduce oxidative stress, and limit cytokine production [231]. Furthermore, urolithin A preserves BBB integrity and prevents neuronal apoptosis in ischemic injury models [229]. Similarly, equol, an isoflavone-derived metabolite, provides vasoprotective and neuroprotective benefits by modulating the ERβ receptor [235]. As a result, polyphenol-rich diets, such as the Mediterranean diet, are associated with a lower risk of vascular and mixed dementias, likely due to improved endothelial health and microbiota-driven metabolite production [239].

### 4.13. Anxiety and Mood Regulation

Microbiota-derived polyphenol metabolites influence emotional regulation and stress resilience. PVLs and dihydroresveratrol have anxiolytic and antidepressant-like effects in preclinical models. They improve behavioral test performance and normalize HPA axis activity, shown by reduced corticosterone levels [230,240]. These compounds increase the expression of GABA receptor subunits in the amygdala and hippocampus, thereby supporting inhibitory neurotransmission and enhancing stress resilience [241,242]. These metabolites also restore gut microbial diversity, reduce dysbiosis-related inflammation, and modulate neuroactive compounds such as tryptophan derivatives and short-chain fatty acids (SCFAs) [243,244,245]. Together, these findings suggest that the microbial products of catechins and resveratrol act through mechanisms that include modulation of the HPA axis, reinforcement of inhibitory neurotransmission, and gut–brain anti-inflammatory signaling, thereby helping to alleviate anxiety- and mood-related disturbances. While preclinical data are robust, human studies are limited, highlighting the need for biomarker-driven clinical trials.

### 4.14. Key Mechanisms of Neuroprotection by Polyphenol-Derived Metabolites

The neuroprotective effects of polyphenol-derived microbial metabolites are mediated through several mechanisms. These include inhibition of protein aggregation, restoration of mitochondrial quality control, attenuation of neuroinflammation, and reinforcement of antioxidant defenses.

− Mitochondrial Biogenesis and Autophagy Activation. Mitochondrial impairment and defective autophagy render neurons vulnerable, leading to energy failure and the formation of dysfunctional organelles. Polyphenol metabolites modulate mitochondrial quality control. Urolithin A activates PINK1/Parkin mitophagy to remove damaged mitochondria and stimulates PGC-1α-driven biogenesis, boosting ATP and reducing ROS. Increased LC3-II, BNIP3, and TFEB indicate greater lysosomal–autophagic flux. These effects prevent neuronal energy failure, key in early neurodegeneration [95,246].
− Inhibition of Amyloid and Protein Aggregation. Protein misfolding and aggregation are pivotal in the progression of Alzheimer’s disease (AD) and Parkinson’s disease (PD). Accumulation of amyloid-β (Aβ) fibrils or α-synuclein aggregates drives synaptic dysfunction and neuronal loss. Urolithin A Inhibits Aβ1–42 fibrillogenesis, destabilizes preformed fibrils, and reduces their cytotoxicity in neuronal cultures, while attenuating oligomer-mediated synaptic impairment in hippocampal neurons [234,246]. Urolithin B and dihydroresveratrol inhibit α-synuclein aggregation by interfering with hydrophobic interactions within the NAC (non-amyloid component) domain, thereby preventing the formation of toxic oligomers and promoting the proteasomal degradation of misfolded proteins [247,248,249].
− Modulation of neuroinflammation. Chronic neuroinflammation, driven by persistent activation of microglia and astrocytes, contributes to blood–brain barrier disruption and neuronal injury. Phenylacetic acid and phenylpropionic acid derivatives suppress IL-6, TNF-α, and IL-1β production in activated microglia, inhibit NF-κB translocation and MAPK phosphorylation, and promote a shift toward M2-like microglia expressing Arg1 and IL-10. This immunomodulatory effect reduces glial scarring, protects the BBB, and limits bystander neuronal damage [240,250].
− Antioxidant and Redox-Modulating Effects. Oxidative stress is a shared mechanism of neuronal injury in AD, PD, and related disorders. Excessive ROS and RNS damage lipids, proteins, and DNA, disrupting synaptic function. 3,4-Dihydroxyphenylacetic acid (DOPAC) and protocatechuic acid activate the Nrf2/ARE pathway, thereby upregulating HO-1, SOD1, and catalase, which in turn increase intracellular GSH and reduce lipid hydroperoxide accumulation. These metabolites also stabilize the mitochondrial membrane potential (Δψm), lower mtROS production, and preserve synaptic plasticity and neurotransmitter balance [212,247,251,252].

Thus, in addition to their activity against foodborne pathogens, polyphenol-derived metabolites exert multitarget neuroprotective effects, including the following:

✓ Block amyloid and α-synuclein aggregation.
✓ Restore mitochondrial homeostasis through biogenesis and mitophagy.
✓ Reprogram neuroinflammation toward a protective phenotype.
✓ Reinforce endogenous antioxidant and redox-balancing systems.

**Figure 5:**
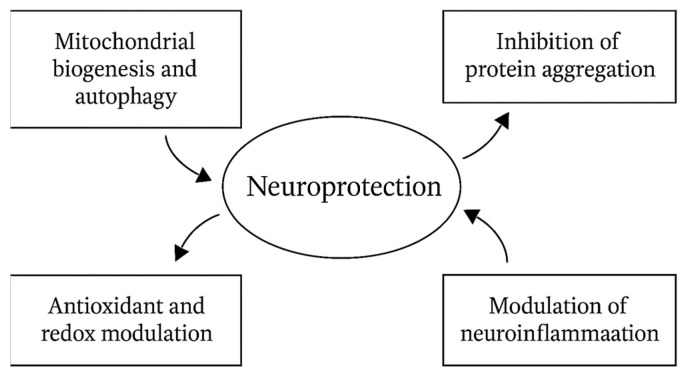
Schematic representation of the main mechanistic pathways through which polyphenol-derived metabolites exert neuroprotective effects. These include inhibition of protein aggregation, enhancement of antioxidant and redox responses, modulation of neuroinflammation, and stimulation of mitochondrial biogenesis and autophagy.

**Figure 6:**
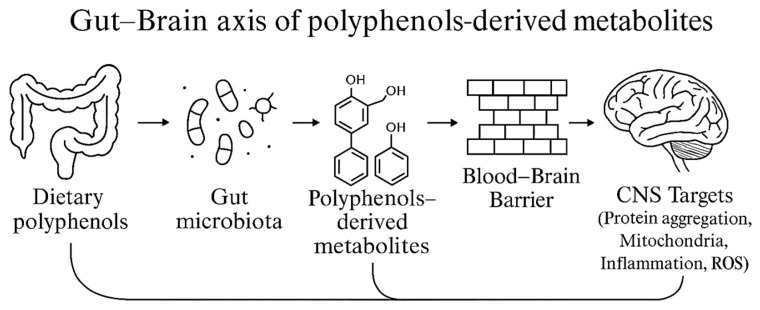
Overview of the gut–brain axis linking dietary polyphenols to brain health. After consuming polyphenol-rich foods, the gut microbiota converts parent compounds into bioactive metabolites (e.g., phenyl-γ-valerolactones, urolithins, dihydroresveratrol, protocatechuic acid, and DOPAC). These metabolites enter circulation, cross the blood–brain barrier, and act on key pathways of neurodegeneration—protein aggregation, mitochondrial dysfunction, neuroinflammation, and oxidative stress—thereby supporting neuroprotection, mood regulation, and reduced cognitive decline.

This pleiotropic action profile underlines their therapeutic potential as next-generation neuroprotective agents in AD, PD, and other neurodegenerative conditions (Table 10, Figure 5 and Figure 6).

## 5. Critical Perspective and Limitations

Despite significant progress in understanding polyphenol metabolism by gut microbiota, the primary research gap is the limited understanding of how different human metabotypes consistently affect long-term health outcomes across populations. Several knowledge gaps persist in current research. The majority of studies employ in vitro assays or simplified microbial models, which fail to adequately represent the complex physicochemical and ecological environment of the human gut and its interactions with host tissues [48]. The dynamic processes of microbial biotransformation, instability of intermediate metabolites, and interindividual differences in gut metabotypes further hinder the establishment of causal relationships between dietary intake, metabolite production, and systemic effects [52]. Additionally, the influence of diet, lifestyle, and genetic factors on polyphenol metabolism and metabotype development remains largely unexplored. Furthermore, most studies have been conducted in vitro or using animal models, and additional human clinical trials are needed to validate the findings. Understanding the clinical implications of metabotypes can lead to improved dietary recommendations and therapeutic interventions tailored to individual metabotypes. Potential clinical applications include using metabotype information to enhance the efficacy of polyphenol-based supplements and designing personalized nutrition plans that optimize the health benefits of consuming polyphenol-rich foods. More research in these areas will facilitate the translation of basic science findings into actionable clinical practices. Analytical limitations also remain, as many low-molecular-weight phenolic metabolites are present at nanomolar concentrations and are challenging to detect or quantify accurately in biological fluids [253]. Additionally, most experimental designs do not assess multiple endpoints, such as anti-biofilm activity, gut barrier modulation, and neuronal protection, within a unified framework. This lack of integration limits a comprehensive understanding of their pleiotropic functions. To improve translational relevance, standardization of experimental protocols, adoption of advanced multi-omics approaches, and use of microfluidic and organoid-on-chip models that simulate the gut–brain axis are required [254,255]. Ultimately, rigorously controlled human intervention studies are necessary to confirm the physiological significance of microbial-derived polyphenol metabolites and to elucidate their dose–response relationships in vivo [51].

## 6. Conclusions and Future Directions

To advance the field, future research must address the experimental and methodological limitations previously outlined and engage directly with the practical translation of mechanistic knowledge into clinical and nutritional frameworks. Specifically, investigations should prioritize elucidating the host and microbial factors that modulate the metabolism and bioactivity of dietary polyphenols. Given that gut microbiota converts polyphenols into a range of low-molecular-weight metabolites—including urolithins, valerolactones, phenolic acids, and dihydroresveratrol—which possess greater stability, enhanced bioavailability, and increased biological activity compared to their parent compounds, it is critically important to delineate the specific microbiota profiles and metabolic pathways responsible for these transformations. This knowledge will inform the design of personalized nutritional strategies and interventions, such as microbiota-informed dietary recommendations that consider an individual’s metabotype, genetic background, and habitual dietary intake. Moreover, future studies should rigorously evaluate the efficacy of innovative delivery systems, including synbiotic formulations, nanoencapsulated polyphenols, and targeted delivery mechanisms, in optimizing colonic bioavailability and therapeutic outcomes in both infectious and neurodegenerative conditions. By focusing on the pathways and clinical implications of polyphenol-derived metabolites, research can provide robust evidence to guide the development of next-generation nutraceuticals and adjunct therapies. This approach holds significant potential to address urgent health concerns such as antimicrobial resistance, gut dysbiosis, and neurodegenerative diseases, ultimately facilitating the integration of polyphenol research into precision medicine and public health policy.

## Figures and Tables

**Table 1:** Main classes of dietary polyphenols: representative compounds, food sources, and key features.

Polyphenol Class	Representative Compounds	Main Dietary Sources	Key Features
Phenolic acids	gallic acid, protocatechuic acid, caffeic acid, ferulic acid	berries, coffee, tea, whole grains, olives	Widely distributed in plants; precursors of many microbial metabolites [11]
Flavonoids	quercetin, catechins (EGCG), naringenin, hesperetin, anthocyanins	fruits (apples, berries, citrus), vegetables, tea, cocoa, red wine	Largest subclass; diverse structures and bioactivities [12]
Stilbenes	resveratrol, piceatannol	grapes, red wine, peanuts, berries	Known for their antioxidant and neuroprotective effects [13]
Lignans	secoisolariciresinol, matairesinol, enterolactone	flaxseed, sesame, whole grains, and vegetables	Converted by gut microbiota into enterolignans [14]
Tannins (hydrolyzable & condensed)	ellagitannins, proanthocyanidins	Nuts, berries, pomegranate, tea, wine	High molecular weight; precursors of urolithins and valerolactones [15]

**Table 2:** Main flavonoid subclasses, including the key biological activities, bioavailability, and metabolism.

Flavonoid Subclass	Representative Compounds	Main Dietary Sources	Key Biological Activities	Bioavailability and Metabolism
Flavonols	Quercetin, Kaempferol	Onions, kale, apples, berries	Potent antioxidant and anti-inflammatory effects	Mostly occur as glycosides; poorly absorbed in the small intestine but extensively metabolized by gut microbiota into bioactive phenolic acids.
Flavones	Luteolin, Apigenin	Parsley, celery, chamomile	Anti-cancer and neuroprotective properties	Undergo microbial deconjugation and transformation into more minor phenolic metabolites with enhanced bioactivity.
Flavanones	Naringenin, Hesperetin	*Citrus* fruits (oranges, grapefruits)	Vascular protection, antioxidant, and anti-inflammatory effects	Microbial metabolism yields phenylpropionic and phenylacetic acids, which support cardiovascular health.
Flavanols (Catechins)	Epicatechin, Epigallocatechin gallate (EGCG)	Tea (especially green tea), cocoa, grapes	Modulation of gut microbiota; cardiovascular and metabolic benefits	Converted into valerolactones and hydroxyphenylvaleric acids by colonic microbiota, and exhibit improved absorption and stability.
Anthocyanins	Cyanidin, Delphinidin	Berries, grapes, red cabbage, eggplants	Anti-inflammatory, anti-diabetic, neuroprotective activities	Rapidly degraded by gut microbiota to produce phenolic acids with preserved bioactivity.
Isoflavones	Genistein, Daidzein	Soybeans, legumes	Estrogenic, anti-osteoporotic, and cardioprotective effects	Act as phytoestrogens; metabolized by intestinal bacteria (e.g., into equol) with enhanced bioavailability and selective estrogen receptor modulation.

**Table 3:** Principal factors affecting polyphenol content and its subsequent biological implications.

Factor Influencing Polyphenol Content	Description	Biological Implication
Processing	Drying, thermal treatment, fermentation, and mechanical processing can degrade, tianstorm or release polyphenols from plant	Alters structure and bioactivity, affects colonic availability
Ripeness and harvest timing	Levels of anthocyanins, flavan-3-ols, and flavonols vary significantly with ripening, with optimum harvest being matrix-dependent	Modulates precursor availability for colonic bioconversion
Agricultural and environmental conditions	Organic practices; UV exposure, altitude, and water availability con modify, polyphenol biosynthesis via stress responses	Affects content and profile of phenctic compounds in crops

**Table 4:** Microbial pathways and enzymes involved in polyphenol metabolism.

Polyphenol Class	Microbial Transformation Pathways	Key Enzymes	Representative Metabolites
Flavan-3-ols	Ring fission, dehydroxylation, and decarboxylation	Reductases, dehydroxylases, esterases	5-(3′,4′-Dihydroxyphenyl)-γ-valerolactone; Hydroxyphenylpropionic acids [46,47,48,49]
Ellagitannins	Hydrolysis, lactonization	Tannase, decarboxylases	Urolithins (A, B, C, D) [44,50]
Flavonols	Deglycosylation, dehydroxylation	β-Glucosidases, reductases	Phenylacetic acids, phenylpropionic acids [51,52,53]
Anthocyanins	Deglycosylation, ring cleavage	β-Glucosidases, esterases	Protocatechuic acid, gallic acid [21,53,54]
Stilbenes	Hydrogenation, dehydroxylation	Reductases, dehydroxylases	Dihydroresveratrol, lunularin [54]
Lignans	Demethylation, dehydroxylation, dehydrogenation	β-Glucosidases, dehydrogenases	Enterodiol, enterolactone [55,56].
Tannins (general hydrolysable)	Hydrolysis, microbial fermentation	Esterases, decarboxylases	Gallic acid, pyrogallol, catechol derivatives [57]

**Table 5:** The main classes of microbial metabolites, the dominant microbial taxa involved, and their functional health effects.

Microbial Metabolites	Main Microbial Genera	Functional Outcomes
Urolithins A, B, C, D	*Gordonibacter*, *Ellagibacter*, *Akkermansia*	Anti-inflammatory, anti-biofilm, mitochondrial biogenesis [37,62,63,64]
γ-Valerolactones, hydroxyvaleric acids	*Clostridium*, *Eubacterium*, *Blautia*, *Flavonifractor plautii*, *Eggerthella*, *Lactobacillus*	BBB modulation, QS inhibition, redox regulation [33,65,66,67]
Protocatechuic acid, hippuric acid, phloroglucinol	*Bifidobacterium*, *Lactobacillus*, *Bacteroides*	Antioxidant, barrier protection, microbial modulation [68,69,70]
Dihydroresveratrol, lunularin	*Eggerthella lenta*, *Slackia equolifaciens*, *Adlercreutzia*	Anti-amyloidogenic, estrogenic modulation, neuroprotective, antifungal [43,54]
Hydroxyphenylpropionic acids, benzoic acids	*Faecalibacterium*, *Roseburia*, *Anaerostipes*	SCFA co-production, TLR modulation, colonocyte health [71,72,73,74]
Equol, O-desmethylangolensin	*Slackia*, *Adlercreutzia*, *Eggerthella*	Estrogen receptor modulation, antioxidant, neuroprotection [75,76,77]
Enterodiol, enterolactone	*Bacteroides*, *Ruminococcus*, *Clostridium*, *Eggerthella*	Antiproliferative, estrogenic/anti-estrogenic, cardioprotective, antioxidant, cardioprotective [71,78,79,80]
3,4-dihydroxyphenylacetic acid, phenylacetic acid	*Eubacterium*, *Lactobacillus*, *Bacteroides*	Anti-inflammatory, immune modulation, antioxidant, intestinal protection [52,81,82,83]
8-prenyl-naringenin	*Eubacterium limosum*	Estrogen modulation, antioxidant activity [84,85]
Tetrahydrocurcumin, dihydrocaffeic acid	*Escherichia coli*, *Blautia*, *Clostridium*	Anti-inflammatory, immune modulation, antioxidant [59,86]
Caffeic acid, ferulic acid	*Bifidobacterium*, *Lactobacillus*, *Eubacterium*	Glycemic modulation, antioxidant, liver protection [87,88,89]
3-(4-hydroxyphenyl) propionic acid	*Clostridium*, *Eubacterium*, *Bacteroides*	Anti-inflammatory, lipid modulation, cardiovascular protection [44,86]
Gallic acid, ellagic acid	*Lactobacillus*, *Bifidobacterium*, *Streptococcus*	Antimicrobial, immune modulation, gut protection [45,90]
Phenylpropionic acid, phenylacetic acid	*Bacteroides*, *Clostridium*, *Eubacterium*	Antioxidant, microbiota modulation, cardiovascular protection [52,91,92]
3,4-dihydroxyphenylacetic acid, protocatechuic acid	*Bacteroides*, *Clostridium*, *Eubacterium*	Anti-inflammatory, immune modulation, intestinal protection [21,48,83,90]

**Table 6:** Summary of key mechanisms underlying polyphenol–microbiota interactions, highlighting microbial transformation, selective modulation of gut taxa, functional gene induction, feedback loops, and downstream immunometabolic effects.

Aspect	Mechanism	Examples
Microbial metabolism of polyphenols	Microbial enzymes degrade polyphenols into more minor, bioactive metabolites	β-glucosidases, esterases, reductases; production of urolithins, valerolactones, hydroxyphenylacetic acids. [46,48]
Microbiota modulation by polyphenols	Selective growth promotion of beneficial microbes and suppression of pathogens	↑ *Akkermansia muciniphila*, *Faecalibacterium prausnitzii;* ↓ *Clostridium* spp., Enterobacteriaceae [52,60,61,101,102,103,104]
Functional gene enrichment	Polyphenol intake increases the abundance of microbial genes involved in polyphenol catabolism.	Tannase, phenolic acid decarboxylase genes [45,104]
Feedback amplification of biotransformation	Polyphenol-induced taxa enhance further degradation of polyphenols into more minor metabolites.	Increased production of urolithins and hydroxycinnamic acid derivatives with repeated intake [21,46]

**Table 7:** Mechanistic anti-biofilm actions of polyphenol-derived molecules, Abbreviations: QS, quorum sensing; EPS, extracellular polymeric substances; AHL/AIP, acyl-homoserine lactone/autoinducing peptide; PVLs, phenyl-γ-valerolactones; DOPAC, 3,4-dihydroxyphenylacetic acid; T3SS, type III secretion system.

Class	Mechanism	Anti-Biofilm Outcomes
Urolithins (A/B)	QS interference; antivirulence at sub-MIC	↓ AHLs, ↓ motility, ↓ biofilm maturation; reduced toxin expression (e.g., *C. difficile*) [134,184,185]
PVLs & hydroxyphenylvaleric acids	Anti-adhesive; QS attenuation	↓ adhesion to bladder cells; ↓ initial attachment; putative repression of virulence in enterics [42]
DOPAC, 3,4-DHPPA, 3-HPA	Anti-adhesive; antibiotic sensitization	↓ adhesion (UPEC, *Salmonella*); ↑ susceptibility to novobiocin; virulence attenuation [81,177]
Polyphenol metabolites (general)	Membrane & macromolecule disruption	Membrane depolarization; ROS-mediated damage; cell lysis (strain-dependent) [137,138,139,140,142]
EGCG and galloylated flavonoids	Anti-amyloid (biofilm matrix); membrane effects	Off-pathway oligomers; weakened matrix; ↓ biomass; ↑ antibiotic susceptibility [104,144]
Surface-active metabolites/coatings	Physical interference with adhesion/EPS	↑ electrostatic repulsion; ↑ hydrophilicity; ↓ initial attachment [145]
Combinations (polyphenols-derived metabolites + antibiotics)	Synergy/sensitization	↓ MIC/MBC; restored activity vs. resistant biofilms; ↓ selective pressure [170,179,180]

**Table 8:** Summary of the foodborne pathogen-focused evidence and polyphenols-derived metabolites actions, and specific readouts (adhesion, QS, EPS, virulence, antifungal morphogenesis). Where mechanisms are inferred from close analogues (e.g., catechin → valerolactone), this is noted in text.

Pathogen	Polyphenols	Readouts
Uropathogenic *E. coli* (UPEC), *E. coli* O157	PVLs; DOPAC; phenolic acids	↓ adherence to T24 cells; dose-dependent anti-adhesion [49,94,152]
*S. aureus*/MRSA	EGCG, tannic acid; phenolics	↓ biofilm maturation; ↑ IsaA; ↑ antibiotic efficacy [94,144].
*S. enterica*	Catechin-derived metabolites (PVLs, HPVAs) (inference); EGCG	↓ biofilm; ↓ virulence; synergy with ciprofloxacin (in vivo) [153,155]
*C. sakazakii*	Coenzyme Q0; chlorogenic acid	↓ adhesion/motility; biofilm disruption [161]
*L. monocytogenes*	Quercetin; EGCG; general phenolics	↓ adhesion/invasion; ↓ hemolysis; ↓ biofilm [167,168,169]
General AMR context	polyphenols-derived metabolites + antibiotics	Enhanced efficacy vs. biofilms; ↓ resistance pressure [179,180,183]

**Table 9:** List of some pathogens involved in CNS and neurodegenerative diseases.

Bacterium	Effect on CNS/Neurodegenerative
***E. coli*** ** (EHEC, K1)**	Neuroinflammation, oxidative stress, and neuronal injury. Contributes to Alzheimer’s (amyloid-β accumulation) and Parkinson’s (α-synuclein aggregation) [186,202,203,204,213,214,215,217,221,223,226]
***S. enterica***	Cognitive decline and neurodegeneration due to chronic inflammation. May exacerbate Alzheimer’s- or Parkinson-like pathology through endotoxin exposure and α-synuclein aggregation [205,206,215,216,218,219,223]
***L. monocytogenes***	Causes meningitis, meningoencephalitis, encephalitis. Leads to chronic neuroinflammation, neuronal degeneration, and BBB disruption [199,207,211,218,219]
***C. botulinum***	Neurotoxicity, neuromuscular paralysis, and possible contribution to chronic neurodegenerative conditions (by altering gut–brain axis). [208,212]
***Streptococcus suis***	Induces bacterial meningitis and neuroinflammation [220]
***C. jejuni***	Associated with Guillain–Barré Syndrome (GBS) and potentially with multiple sclerosis (MS)-like pathology [224]

**Table 10:** Neuroprotective Mechanisms of Polyphenol-Derived Metabolites.

Metabolite/Class	Mechanism of Action
Urolithin A (UA)	Inhibition of amyloid aggregation; synaptic protection [252]
Phenyl-γ-valerolactones (PVLs)	Proteostasis modulation; anti-amyloid oligomer detoxification [228,232]
Urolithin A (UA)	Mitophagy activation & mitochondrial biogenesis; BBB support [95,229,246]
Microbial phenolic acids (3,4-dihydroxyphenylacetic acid; protocatechuic; dihydrocaffeic; conjugates)	Anti-inflammatory & antioxidant actions [231]
Phenylacetic & phenylpropionic acid derivatives	Neuroinflammation modulation; microglial polarization [240,250]
DOPAC & Protocatechuic acid	Antioxidant/redox modulation [251]
PVLs & phenolic acids (vascular axis)	Endothelial/BBB support relevant to VCI [231,238]
Equol (microbiome derived ERβ agonist)	Vascular–cognitive aging; metabotype-aware translation [235]

## Data Availability

No new data were created or analyzed in this study. Data sharing is not applicable to this article.
